# PPARγ and PGC-1α as Therapeutic Targets in Parkinson’s

**DOI:** 10.1007/s11064-014-1377-0

**Published:** 2014-07-10

**Authors:** Juan Carlos Corona, Michael R. Duchen

**Affiliations:** Department of Cell and Developmental Biology, University College London, London, WC1E 6BT UK

**Keywords:** PPAR gamma, PGC-1 alpha, Parkinson’s disease, Neuroprotection

## Abstract

The peroxisome proliferator-activated receptor gamma (PPARγ) is a ligand-activated transcriptional factor that belongs to the nuclear hormone receptor superfamily. PPARγ was initially identified through its role in the regulation of glucose and lipid metabolism and cell differentiation. It also influences the expression or activity of a number of genes in a variety of signalling networks. These include regulation of redox balance, fatty acid oxidation, immune responses and mitochondrial function. Recent studies suggest that the PPARγ agonists may serve as good candidates for the treatment of several neurodegenerative disorders including Parkinson’s disease (PD), Alzheimer’s disease, Huntington’s disease and amyotrophic lateral sclerosis, even though multiple etiological factors contribute to the development of these disorders. Recent reports have also signposted a role for PPARγ coactivator-1α (PGC-1α) in several neurodegenerative disorders including PD. In this review, we explore the current knowledge of mechanisms underlying the beneficial effects of PPARγ agonists and PGC-1α in models of PD.

## Introduction

The peroxisome proliferator-activated receptors (PPARs) are ligand-inducible transcription factors that belong to the hormone nuclear receptor superfamily. They are involved in the transcriptional control of genes regulating various physiological processes such as lipid homeostasis, glucose metabolism, inflammation, cellular differentiation and proliferation [1, 2]. PPARs act mainly as lipid sensors, regulating metabolism in response to dietary lipid intake and direct the subsequent metabolism and storage of lipids [3]. Three isoforms have been identified, PPARα, PPARβ/δ, and PPARγ. These three isoforms differ in terms of their tissue distribution, ligand specificity and physiological role. PPARα acts primarily to regulate energy homeostasis through its ability to stimulate the breakdown of fatty acids and cholesterol, driving gluconeogenesis and reduced triglyceride levels. This receptor in particular acts as a lipid sensor, binding fatty acids and initiating their subsequent metabolism. The PPARβ/δ receptors bind and respond to VLDL-derived fatty acids, eicosanoids, including prostaglandin A1, and are involved in fatty acid oxidation. PPARγ stimulates adipocyte differentiation and lipid metabolism. PPARγ operates in the metabolism of lipid and carbohydrate metabolism and its activation is related to reduction of glucose levels [4].

Peroxisome proliferator-activated receptors (PPARs) are activated by small, lipophilic compounds and regulate gene expression by forming heterodimers with retinoid-X-receptors. Once activated the PPAR/retinoid-X-receptors heterodimer binds to the specific DNA sequence [peroxisome proliferator response element (PPRE)] on the promoter region of PPAR target genes [2, 5] to modulate transcriptional activity. The activity of PPARs is also regulated by posttranslational modification such as phosphorylation and sumoylation [6, 7]. For example, there are several mechanisms involved in PPARγ inactivation. Thus, phosphorylation can negatively or positively affect PPARγ activity depending on which specific protein residue is modified [8–11]. The PPARγ activity is decreased via the ubiquitination degradation pathway [12]. Alternatively, PPARγ sumoylation promotes the repression of inflammatory or adipocyte differentiation genes [6, 13].

Peroxisome proliferator-activated receptor alpha (PPARα) ligands include fibrates that are commonly used for the treatment of hypertriglyceridemia and WY14,643 and GW7647. PPARβ/δ ligands include the prostacyclin PGI2, and synthetic compounds GW0742, GW501516, and GW7842. All PPARs can be activated by polyunsaturated fatty acids with different affinities [14, 15]. Naturally occurring PPARγ ligands include long chain fatty acids, other natural lipid ligands, eicosanoids and the prostaglandin 15d-PGJ2, but also few nonsteroidal antiinflammatory drugs, as ibuprofen, fenoprofen, and indomethacin A [15–17]. Synthetic thiazolidinediones (TZDs), including pioglitazone and rosiglitazone were originally designed as PPARγ agonists and are currently in clinical use as insulin-sensitizing agents for the treatment of type 2 diabetes [15, 18].

## Distribution of PPARs

Peroxisome proliferator-activated receptor alpha (PPARα) is highly expressed in metabolically active tissues, such as liver, kidney, intestine, heart, skeletal muscle, adrenal gland and pancreas during foetal development of rodents [19, 20]. In adult rodent organs, the distribution of PPARα is similar to its foetal pattern of expression. In the central nervous system (CNS), PPARα is expressed at very low levels predominantly in astrocytes and PPARα is most highly expressed in tissues that catabolise fatty acids, such as the adult liver, heart, kidney, large intestine and skeletal muscle [21]. PPARβ/δ is the most abundant in the CNS, PPARβ/δ is expressed ubiquitously in virtually all tissues and earlier during foetal development. PPARβ/δ mRNA is present ubiquitously, with a higher expression in the digestive tract and placenta [19–21]. In the CNS PPARβ/δ is preferentially found in the cerebellum, brain stem and cortex, was enriched in the dentate gyrus/CA1 region and was found in immature oligodendrocytes. Its activation promotes differentiation, myelin maturation and turnover [22, 23]. PPARγ receptors are distributed in several cell types and tissues. Given the role of PPARγ in regulating glucose and lipid metabolism, in promoting lipid storage and adipocyte differentiation [24–26], PPARγ is expressed in white and brown adipose tissue and in the CNS during foetal development of rodents. PPARγ is abundantly expressed in white adipose tissue, and is present at lower levels in skeletal muscle, heart and liver [19–21]. In the CNS, PPARγ is expressed in several cell types including neurons, astrocytes, oligodendrocytes and microglia [16, 26–28]. In neurons, PPARγ immunoreactivity appears mainly as a nuclear labeling although sometimes cytoplasmic staining is detectable in some cortical neuron [28]. High levels of PPARγ have been found in the piriform cortex and olfactory tubercle, in the basal ganglia, in rhomboid, centromedial, and parafascicular thalamic nuclei, in the reticular formation, and in the stellate cells of cerebellar cortex [28]. PPARγ is expressed in the basal ganglia, and in areas expressing dopamine receptors. PPARγ is expressed in adult cultured cortical astrocytes [27, 28].

## PPARγ in Models of Parkinson´s Disease

**Fig. 1:**
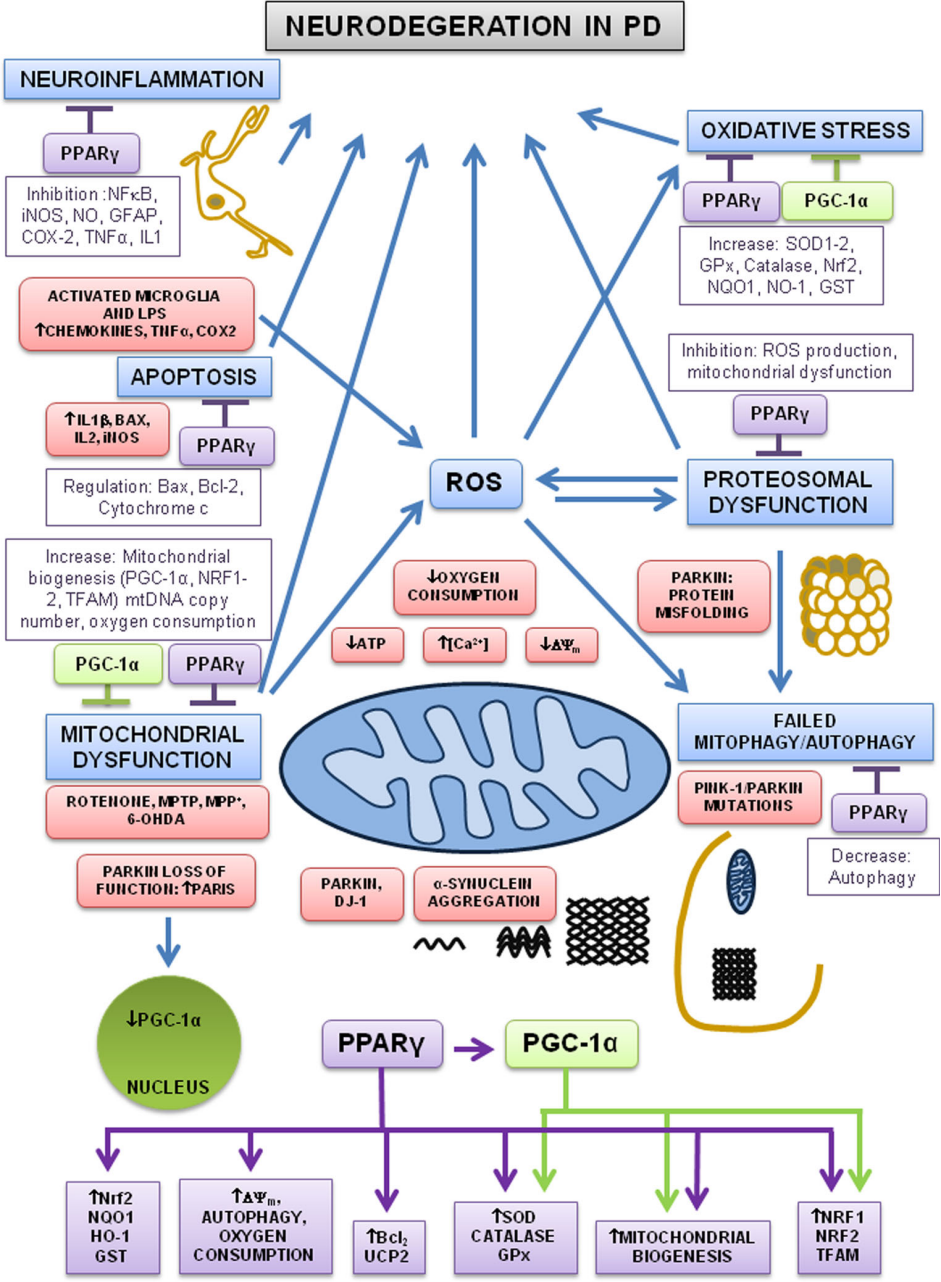
Pathways involved in neuroprotection by PPARγ and PGC-1α in PD. Mitochondrial dysfunction, oxidative stress, proteosomal dysfunction, neuroinflammation, autophagy and apoptosis are all implicated in the pathogenesis of PD. Environmental factors and toxins (rotenone, MPTP, MPP^+^ and 6-OHDA) directly induce both oxidative stress and mitochondrial dysfunction. Different toxins increase oxidative stress (ROS) and cause mitochondrial dysfunction, both increase [Ca^2+^], decrease ATP, decrease mitochondrial membrane potential, decrease oxygen consumption and cause failure in autophagy, proteosomal dysfunction and abnormal protein aggregation which ultimately lead to neuronal death. Activated microglia release inflammatory cytokines and increase ROS, driving neuronal degeneration. DJ-1 and PARKIN mutations cause aggregation of α-synuclein and PARKIN/PINK-1 mutations cause failure in autophagy. Mutations in the PARKIN gene cause protein misfolding. Mutations in PARKIN also increase expression of the PARIS, thereby repressing the expression of PGC-1α. PPARγ agonists inhibit microglial activation and reduce inflammation by decreasing expression of cytokines, TNF-α, COX2 and iNOS. PPARγ agonists reduced apoptosis by inhibition of BAX, IL2, IL1β and by increasing Bcl-2 expression. PPARγ agonists increase antioxidant defences, mitochondrial biogenesis, oxygen consumption, mitochondrial membrane potential, autophagy, PGC-1α and other transcription factors. Moreover, PGC-1α induces the expression of downstream target genes involved in mitochondrial biogenesis, transcription factors and antioxidant defences. Thus, PGC-1α and PPARγ agonists regulate the expression of several target genes involved in neuronal survival and neuroprotection by inhibiting mitochondrial dysfunction, oxidative stress, proteosomal dysfunction, autophagy, neuroinflammation and apoptosis

Parkinson’s disease (PD) is a chronic neurodegenerative disorder characterized by the progressive loss of dopaminergic neurons of the substantia nigra pars compacta, resulting in deficiency of nigrostriatal dopamine transmission. One pathological feature of the disease is the presence of Lewy bodies that are intraneuronal proteinaceous cytoplasmic inclusions, which include α-synuclein, ubiquitin, and neurofilaments, and are found in all affected brain regions. The basic characteristics of PD include tremor, rigidity, bradykinesia and impaired balance. PD occurs most commonly as a sporadic form (95 %), while familial forms make up the remainder, involving mutations in an array of proteins that include PINK1, PARKIN, LRRK2, fbxo-7 and DJ-1 [29], although environmental factors such as chemicals, pesticides and metals may increase the risk of developing PD [30–32]. Currently there is no effective treatment that slows the progression of the disease, and management remains symptomatic. Although the specific pathomechanism of PD is still unclear, there is ever growing evidence suggesting the involvement of mitochondrial dysfunction, oxidative stress, protein dysfunction, apoptosis, autophagy and chronic neuroinflammation. In recent years, the neuroprotective effects of PPARγ agonists has been assessed in several in vitro and in vivo models of several neurodegenerative conditions including PD [33, 34], Alzheimer’s disease [35, 36], cerebral ischemia [37] and amyotrophic lateral sclerosis [38]. The potential mechanisms of neuroprotection by PPARγ agonists in PD are summarised in (Fig. 1).

1-methyl-4-phenyl-1,2,3,6-tetrahydropyridin (MPTP) administration has been widely used in animals to selectively target dopaminergic neurons and so reproduce PD symptoms [39]. In the acute MPTP model in the rodent, the PPARγ agonist pioglitazone blocked dopaminergic neurodegeneration and reduced astrocytic and microglial activation. However, pioglitazone treatment did not alleviate MPTP-induced loss of tyrosine hydroxylase in the striatum and had only partially protective effects on the MPTP-induced decline in striatal tissue levels of dopamine [40]. In another study pioglitazone was shown to protect against chronic MPTP-induced neurotoxicity, with reduced activation of microglia, reduced induction of iNOS-positive cells and fewer glial fibrillary acidic protein (GFAP) positive cells in both striatum and substantia nigra [41]. Recently it has also been shown that pioglitazone protected against MPTP induced neurotoxicity by the inhibition of monoamine oxidase-B in the striatum. Therefore, blocking the conversion of MPTP to its active toxic metabolite MPP^+^, via inhibition of monoamine oxidase-B [42]. Treatment with rosiglitazone in the chronic MPTP (plus probenecid) mouse model, completely prevented motor and olfactory dysfunction and loss of dopaminergic neurons in the substantia nigra. Rosiglitazone partially protected against loss of striatal dopamine, whereas decreases in DOPAC and dynorphin mRNA in the striatum were completely abolished. Also astrogliosis and number of activated microglia were reduced as assessed by GFAP and CD11b immunostaining, respectively, without affecting MPTP metabolism [43]. In the same model of MPTP plus probenecid, treatment with rosiglitazone was also effective in protecting against partial degeneration of the substantia nigra and the decline of striatal dopamine [33]. In a recent study, pioglitazone was also neuroprotective and antiinflammatory in an MPTP model in the rhesus monkey, with a significant improvement in a clinical rating score. Behavioral recovery was associated with preservation of nigrostriatal dopaminergic markers and reduced infiltration by CD68-positive macrophages in the nigrostriatal area [44]. More recently, the administration of a non-TZD partial PPARγ agonist, was again shown to be neuroprotective in MPTP-induced neurodegeneration, associated with downregulation of neuroinflammation, decreased oxidative stress, and modulation of PPARγ and PPARγ coativator-1α (PGC-1α) expression [45].

Intrastriatal injection of lipopolysaccharide (LPS) in rats has also served to model degeneration of dopaminergic neurons in PD. In this model pioglitazone prevented the loss of dopaminergic neurons and the decline in striatal dopamine levels. Pioglitazone normalized COX-2 expression and increased the expression of uncoupling protein 2 (Uncoupling protein 2 is one of five acknowledged uncoupling proteins and it is located in the inner mitochondrial membrane, where it helps reduce the proton gradient. Also, uncoupling protein 2 may be involved in PD) and increased the expression of mitoNEET, while iNOS induction and oxidative stress were reduced [46, 47]. In dopaminergic neuron-glial cultures, pioglitazone protected neurons from LPS by inhibiting abnormal microglial activation, interfering with phosphorylation of Jun N-terminal kinase and nuclear factor kappa-B, and by suppressing cyclooxygenase-2 expression and the subsequent prostaglandin E(2) synthesis [48]. Pioglitazone also protected dopaminergic neurons against LPS damage by inhibiting iNOS expression and nitric oxide generation by differential regulation of p38 mitogen-activated protein kinase and the phosphoinositide 3-kinase/protein kinase B pathway [49]. Microglial activation has been implicated in the pathogenesis of PD and is believed to aggravate neuronal injury [33, 50]. The anti-inflammatory actions of rosiglitazone against LPS were mediated by its ability to increase IL-4 expression [51]. Thus, production of pro-inflammatory cytokines has been described in a 6-hydroxydopamine (6-OHDA) model of PD, where microglial activation was observed [52]. A recent study demonstrated that pioglitazone did not exert any protection in the 6-OHDA model. The lack of effect of pioglitazone in this model was attributed to the severity of the damage caused by 6-OHDA. However, pioglitazone protected against neuronal loss and motor behaviour in the acute MPTP model [53]. In the 6-OHDA-lesioned rat, the activation of PPARγ receptors by rosiglitazone significantly attenuated the production of both COX-2 and TNF-α expression and increased GFAP expression in the striatum [54].

It has been demonstrated that PPARγ has actions on mitochondrial function. PPARγ activation increased mitochondrial membrane potential and protected cells from apoptosis following growth factor withdrawal [55]. Pioglitazone also increased neuronal glucose uptake and restored brain ATP levels [56, 57]. Pioglitazone increased mitochondrial DNA content, oxygen consumption, PGC-1α and mitochondrial transcription factor A (TFAM) in human adipose tissue and in the neuronal-NT2 cell line [58–61]. Rosiglitazone induced both mitochondrial biogenesis and glucose utilization in mouse brain [62]. In addition, Pioglitazone stabilizes MitoNEET, an iron-sulfur containing outer mitochondrial membrane protein which regulates oxidative capacity [63–65].

Rotenone is a complex I inhibitor and has been widely used to model PD [39, 66]. Consequently, pioglitazone protected against the reduction of locomotor activity and decline in striatal dopamine levels induced by rotenone [67]. In a recent study, it was found that rotenone irreversibly decreased mitochondrial mass, membrane potential and oxygen consumption, while increasing free radical generation and autophagy in human differentiated SH-SY5Y cells. Similar changes were seen in PINK1 knockdown cells, in which the membrane potential, oxygen consumption and mitochondrial mass were all decreased. In both models, all these changes were reversed by treatment with rosiglitazone, which increased mitochondrial biogenesis, increased oxygen consumption and suppressed free radical generation and autophagy [68]. Rosiglitazone significantly increased the expression of proteins related with antioxidant defences and mitochondrial biogenesis (SOD1, Nuclear factor (erythroid-derived 2)-like 2 (Nrf2), NAD(P)H:quinone oxidoreductase 1 (NQO1), PGC-1 and TFAM). Thus, rosiglitazone was neuroprotective in two different models of mitochondrial dysfunction associated with PD through a direct impact on mitochondrial function [68]. Nrf2 is a pivotal upstream transcription factor responsible for the regulation of redox balance. Nrf2 is normally sequestered in the cytoplasm by its inhibitor Keap1. In response to oxidative stress, Nrf2 translocates to the nucleus and dimerizes with another member of the Cap’n’Collar/basic leucine zipper family of transcription factors [69], activating transcription by binding to an antioxidant response element (ARE) located in the promoter of a number of antioxidant genes, including NQO1, Heme oxygenase-1 (HO-1) and Glutathione S-transferase [70, 71]. A number of studies have suggested that Nrf2 and NQO1 protect against cellular dysfunction in different models of PD [72–74]. Recently, it was demonstrated that rosiglitazone increased expression of Nrf2 and the antioxidant enzyme HO-1 acting through the PPARγ-pathway, enhancing elimination of ROS in hepatocytes [75]. The protective effects of TZDs have been attributed also to their antioxidant and anti-apoptotic properties. For that reason, rosiglitazone was shown to protect human neuroblastoma cells against MPP^+^ induced mitochondrial dysfunction by anti-oxidant properties and anti-apoptotic activity via inducing expression of SOD and catalase and regulating the expression of Bcl-2 and Bax and increase the mitochondrial membrane potential [76]. In the MPP^+^ model, rosiglitazone treatment did not alter SOD activity but there was an increase of glutathione S-transferase activity and the protective effects of rosiglitazone were not blocked by the PPARγ antagonist GW9662, suggesting that these effects may be independent of PPARγ activation [77]. Acetaldehyde, an inhibitor of mitochondrial function, causes neuronal death by inducing generation of intracellular reactive oxygen species and cellular apoptosis in human neuroblastoma cells. Rosiglitazone reversed acetaldehyde induced apoptosis by inducing the expression of anti-oxidant enzymes such as SOD and catalase and by regulating expression of Bcl-2 and Bax [78].

## The PPARγ Coativator-1α (PGC-1α)

PPARγ coactivator-1α (PGC-1α) was discovered in brown adipose tissue as a PPARγ coactivator during the thermogenic response to cold [79]. Two other coactivators have been identified, PGC-1β and PGC-1-related coactivator. PGC-1α and PGC-1β display a great degree of homology but are slightly differently regulated [80]. PGC-1α can regulate other nuclear receptors such as the thyroid hormone receptor, the oestrogen receptor, and the oestrogen-related receptor α, aside of acting as a coactivator for PPARs [81]. On the other hand, PGC-1α acts also as a coactivator for other transcription factors such as the nuclear respiratory factors 1 and 2 (NRF-1 and 2), TFAM, myocyte enhancer factor 2, FOXO receptors and hepatic nuclear factor 4 [81]. PGC-1α is highly expressed in tissues with a high-energy demand, such as brown adipose tissue, brain, heart, liver, pancreas, skeletal muscle and kidney [82]. It plays a central role in driving and coordinating mitochondrial biogenesis and respiration, gluconeogenesis and glucose transport, glycogenolysis, fatty acid oxidation, peroxisomal remodeling, muscle fiber-type switching, oxidative phosphorylation and is preferentially expressed in muscle enriched for type I myocytes and can convert the type II myocytes to type I fibers [83]. In addition, PGC-1α also regulates the expression of several ROS detoxifying enzymes, such as SOD1 and 2, catalase and glutathione peroxidase-1 [84]. The activity of PGC-1α is influenced by post-transcriptional modifications, such as protein phosphorylation, acetylation, sumoylation, and methylation [81, 85, 86]. PGC-1α expression can be induced by cold exposure, fasting, and exercise, which require energy expenditure [79, 83, 87]. It has been reported that PGC-1α expression is decreased with aging, possibly owing to decreased sirtuin1 (SIRT1) levels [85] or by the action of p53 that is activated by telomere shortening and suppresses PGC-1α [88]. Drugs such as resveratrol, can act by decreasing PGC-1α acetylation, producing a subsequent increase in PGC-1α activity and its downstream genes [89].

## PGC-1α in PD

The role PGC-1α, which is involved in mitochondrial biogenesis and respiration, has been implicated in PD. As mentioned above, PGC-1α induces the expression of ROS scavenging enzymes (glutathione peroxidase-1, catalase and SOD) and reduces oxidative stress [84]. An increased vulnerability to MPTP induced degeneration of nigral dopaminergic neurons was observed in PGC-1α knockout mice, suggesting a critical role of PGC-1α in neuroprotection. Therefore, Increasing PGC-1α levels dramatically protected neural cells from oxidative stress and cell death [84]. These studies suggested compelling evidence for a role of PGC-1α in neurodegenerative diseases and as a good candidate for the treatment of PD. The mechanisms of neuroprotection by PGC-1α in PD are shown in (Fig. 1).

Activation of PGC-1α increased the expression of nuclear-encoded subunits of the mitochondrial respiratory chain and prevented the dopaminergic neuron loss induced by mutant α-synuclein or the pesticide rotenone in cellular disease models [90]. Also, it has been shown that PGC-1α knockdown increased α-synuclein accumulation and led to down regulation of the AKT/GSK-3β signaling pathway in human neuronal cells [91]. A substrate for PARKIN, the PARKIN-interacting substrate (PARIS), is a zinc-finger protein which is highly expressed in the substantia nigra. PARIS represses the expression of PGC-1α and NRF-1 and the site of interaction between PARIS and PGC-1α is a sequence that is involved in the regulation of insulin responsiveness and energy metabolism. Conditional knockout of PARKIN in adult animals led to progressive loss of dopamine neurons which was dependent on PARIS expression. Moreover, overexpression of PARIS led to the selective loss of dopamine neurons in the substantia nigra, and this was reversed by either PARKIN or PGC-1α coexpression [92]. A recent study reported that PINK1 mutations impair PARKIN recruitment to mitochondria in neurons, increased mitochondrial copy number, and upregulation of PGC-1α [93]. Other studies, have shown that transgenic overexpression of PGC-1α or activation of PGC-1α by resveratrol protect dopaminergic neurons in the MPTP mouse model of PD [94]. Recently it was shown that adenoviral delivery of PGC-1α in the nigrostriatal system increased dopaminergic death [95]. This effect could be the result of excessive overexpression of PGC-1α, resulting in mitochondrial hyperactivity and increased production of ROS. Apparently, the studies related to the role of PGC-1α in PD have provided inconsistent data regarding the effects of PGC-1α activation or overexpression in PD [96].

## Conclusion

In conclusion, a number of molecular pathways including oxidative stress, mitochondrial dysfunction, protein dysfunction, apoptosis, autophagy and neuroinflammation are implicated in the pathophysiology of PD. As currently available drugs cannot slow down the progression of the disease, using a combination of several pharmacological agents may offer better promise for neuroprotection, modulating several molecular pathways involved in the pathophysiology simultaneously. PPARγ agonists and PGC-1α exhibit a wide range of activities that positively influence the pathology of PD in experimental models, and they have the capacity to be neuroprotective by regulating the expression of genes involved in neuronal survival processes. The compelling results from in vitro and in vivo models of PD underline the beneficial effects of PPARγ agonists and PGC-1α for future therapies. Thus, PPARγ agonists and PGC-1α could be valuable potential therapeutic targets for neurodegenerative diseases. Finally, understanding the molecular mechanisms by which PPARγ and PGC-1α exert their neuroprotective effects will be helpful in developing an effective treatment for PD.

## Abbreviations

PD: Parkinson’s disease
PPARs: Peroxisome proliferator-activated receptors
TZDs: Thiazolidinediones
PGC-1α: Peroxisome proliferator-activated receptor gamma coactivator-1 alpha
ROS: Reactive oxygen species
SOD: Superoxide dismutase
NQO1: NAD(P)H:quinone oxidoreductase 1
Nrf2: Nuclear factor (erythroid-derived 2)-like 2
TFAM: Mitochondrial transcription factor A
MPTP: 1-Methyl-4-phenyl-1,2,3,6-tetrahydropyridine
MPP^+^: 1-Methyl-4-phenylpyridinium ion
CNS: Central nervous system
HO-1: Heme oxygenase-1
NRF: Nuclear respiratory factor
6-OHDA: 6-Hydroxydopamine
LPS: Lipopolysaccharide
